# DNA barcoding of *Clarias gariepinus*, *Coptodon zillii* and *Sarotherodon melanotheron* from Southwestern Nigeria

**DOI:** 10.12688/f1000research.7895.1

**Published:** 2016-06-08

**Authors:** Mofolusho O. Falade, Anthony J. Opene, Otarigho Benson

**Affiliations:** 1 Cellular Parasitology Programme, Cell Biology and Genetics Unit, Department of Zoology, University of Ibadan, Ibadan, Nigeria; 2 Department of Biological Science, Edo University, Iyamho, Edo State, Nigeria

**Keywords:** DNA Barcode, Clarias, Tilapia, COI gene, 16S rRNA

## Abstract

DNA barcoding has been adopted as a gold standard rapid, precise and unifying identification system for animal species and provides a database of genetic sequences that can be used as a tool for universal species identification. In this study, we employed mitochondrial genes 16S rRNA (16S) and cytochrome oxidase subunit I (COI) for the identification of some Nigerian freshwater catfish and Tilapia species. Approximately 655 bp were amplified from the 5′ region of the mitochondrial cytochrome C oxidase subunit I (COI) gene whereas 570 bp were amplified for the 16S rRNA gene. Nucleotide divergences among sequences were estimated based on Kimura 2-parameter distances and the genetic relationships were assessed by constructing phylogenetic trees using the neighbour-joining (NJ) and maximum likelihood (ML) methods. Analyses of consensus barcode sequences for each species, and alignment of individual sequences from within a given species revealed highly consistent barcodes (99% similarity on average), which could be compared with deposited sequences in public databases. The nucleotide distance between species belonging to different genera based on COI ranged from 0.17% between *Sarotherodon* *melanotheron* and *Coptodon zillii* to 0.49% between *Clarias gariepinus* and *C. zillii*, indicating that *S. melanotheron* and *C. zillii* are closely related. Based on the data obtained, the utility of COI gene was confirmed in accurate identification of three fish species from Southwest Nigeria.

## Introduction

The use of a globally recognized short DNA sequence, DNA barcode, for identification of species has gained global support as an applicable tool for species identification, particularly with respect to fishes as coordinated by the fish barcode of life (FISH-BOL; www.fishbol.org) ^1^. Fish biodiversity in tropical Africa demonstrate an amazing variety of shape, size, and color. However, many of these fishes are under immense pressure from overfishing and climate change. In addition, the lack of appropriate methods of identification has limited our ability for classification, thus limiting the information available for fishery management ^2^. About 65% of fishes captured worldwide have been identified to species level ranging from about 90% in temperate areas to less than 40% in tropical regions ^3^. However, there is the need to identify more fish species from Africa, where there is a dearth of information on indigenous fish species ^4^. The paucity of taxonomic data on local fish can be ascribed to the limitations imposed by traditional-based morphological identification, which can be confusing and unreliable due to problems of intraspecific, phenotypic and cryptic variation often overlapping among sister taxa in nature ^5^. Consequently, the limitations imposed by morphological identification, have made the use of molecular diagnostic tools as a prerequisite for effective species identification ^6^. DNA-based identification techniques have been developed and shown to be analytically important for characterization of organisms. DNA taxonomic techniques such as DNA barcoding have been useful for species identification and description ^7^. DNA barcoding has been used to identify species and is important in characterizing biological diversity. This technique involves the amplification and sequencing of short universal molecular tags from a highly conserved gene. The mitochondrial cytochrome oxidase I (COI) gene is commonly used for this purpose ^8^. The method is rapid, accurate and useful in delineating differences between species ^9^. Consequently, the mitochondrial genes COI and 16S have been successfully employed in species identification based on DNA barcodes ^10^ and a series of barcoding projects involving various organisms from different geographic regions is available at the public barcode library ( www.barcodinglife.com) ^11^.

Despite the large information that exists for temperate fishes ^12^, there are rather limited data for tropical fishes especially from Nigeria. A report by Nwani and colleagues employed DNA barcoding to discriminate freshwater fishes from Southeastern Nigeria where they provided a river system-level phylogeographic resolution of some of the fishes identified in their study ^13^. Recently, Nwakanma *et al.*, ^14^ also employed DNA barcoding in studying genetic diversity of fishes from Ugwu-omu Nike river of Enugu State, also in Southeastern Nigeria. It is therefore imperative to apply these tools to fishes from other areas in the country. Two of the most common freshwater fishes consumed by the population are from the genus *Clarias* and *Tilapia (Coptodon)* ^15, 16^. Of these, the most utilized in aquaculture and fish farming is *C. gariepinus*. *Tilapia*, belonging to the family Cichlidae, is a highly diverse group of more than 70 species found in Nigeria ^15^. However, interbreeding of these fishes makes species delineation through morphology difficult.

Consequently, in this study, COI and 16S genes were employed in performing an identification and diversity study of *C. gariepinus*, *Coptodon zillii* and *Sarotherodon melanotheron* from Southwestern Nigerian freshwater bodies.

## Materials and methods

### Ethics

Ethical approval for animal experiments is given based on institutional guidelines. Collection of fish specimens and all laboratory experiments were thus performed in strict accordance with the recommendations of the University of Ibadan Ethical Committee on the use of laboratory animals for research.

### Sample sites and collection

Three fish species, *C. gariepinus*, *C. zillii* and *S. melanotheron* were obtained with the aid of a local fisherman from Odooba River and Asejire Lake in Southwestern Nigeria. Both sites are tropical and characterized by two annual seasons of wet (April–September) and dry (October to March) seasons. The former site lies between 3.9°E and 7.4°N close to the University of Ibadan, Oyo State. Dead fish samples were collected and transported on ice to the Hydrobiology and Fisheries Laboratory of the Department of Zoology, University of Ibadan, where all fish specimens were morphologically identified to the species level by fish taxonomists using identification keys described by Olaosebikan and Raji ^15^. Thereafter, fish specimens were preserved at –80°C until DNA extraction.

### DNA extraction

Excised muscle tissue samples from the side of each fish were used to extract DNA. DNA was isolated using the QIAamp ^®^ DNA mini kit (QIAGEN, USA), following the manufacturer’s instructions. The concentrations and purity of the extracted DNA were estimated using a Nanodrop spectrophotometer (Nanodrop ^®^ ND -1000- NanoDrop Technologies, Inc.). Extracted DNA was visualized on a 2% agarose gel stained with ethidium bromide.

### PCR and DNA sequencing

To amplify from the 5 ^/^ region approximately 570 bp fragment of the 16S rRNA gene and 655 bp of the COI gene, PCR reactions were conducted using the following primers: for 16 rRNA, 16SarL-F (5′-CGC CTG TTT ATC AAA AAC AT-3′) and 16SbrH-R (5′-CCG GTC TGA ACT CAG ATC ACG T-3′); for COI, FishF1 (5 ^/^-TCA ACC AAC CAC AAA GAC ATT GG CAC-3 ^/^) and FishR1 (5 ^/^-TAG ACT TCT GGG TGG CCA AAG AAT CA-3 ^/^) ^17^.

Amplification reactions for both genes were carried out in a total reaction volume of 10μL. The 10 μL PCR reaction mixes included 1 X PCR buffer, 5.0 mM MgCl _2_, 0.2 μM of each primer, 0.4 μL of 0.2 units of *Taq* polymerase, 0.25 mM of mixed dNTPs and 100ng of DNA template. The thermal profiles used were as follows: for 16S rRNA gene, initial step at 94°C for 5 minutes followed by 35 cycles of 94°C for 30 s, 53.9°C for 40 s and 72°C for 45 s, and a final step at 72°C for 5 min. For the COI gene, an initial denaturation at 94°C for 5 minutes, 35 cycles of 94°C for 45 seconds, 60°C for 45 seconds and a final step at 72°C for 1 minute, and concluded with a final elongation step at 72°C for 8 minutes followed by a hold at 4°C. PCR products were visualized on a 2% agarose gel stained with ethidium bromide and the most intense products were selected for sequencing. Purified DNA products were labelled using BIG Dye Terminator v.3.1 Cycle Sequencing Kit (Applied Biosystems Inc., CA, USA) with ABI 3130Xl BigDye ^®^ Terminator model following manufacturer’s instructions. The PCR sequencing protocol cycling conditions were as follows: an initial step of 2 minutes at 96°C and 35 cycles of 30 s at 96°C, 15 s at 55°C, and 4 minutes at 60°C.

### Sequence quality control measures

In order to assure the quality and integrity of the fish samples barcoded in this study, all the PCR amplified products and their corresponding DNA sequences were larger than 600 bp. This ensures that no nuclear DNA sequences originating from mt DNA sequences (NUMTs) being amplified as the limit of NUMTs rarely reach 600 bp. Standard nucleotide BLAST (BLASTN) ^18^ and BOLD Identification System were used to compare the sequences and those sequences showing 99–100% alignment with no gaps or indels (insertions/deletions) was selected. The emphasis of these tools is to align regions of sequence similarity with the partial coding sequence of fish mitochondrial COI gene. The sequences for all the specimens were aligned using Clustal W as implemented in MEGA (version 5.2) ^19^.

### Data analysis

The total dataset (32) included 16 COI sequences and 16 16S rRNA sequences for 3 fish species comprising 16 individuals. The sequence similarity search for species identification was done in two public databases, viz., BOLD ( http://www.boldsystems.org/index.php/IDS_OpenIdEngine) and GenBank ( http://blast.ncbi.nlm.nih.gov/Blast.cgi). The highest percent pairwise identity for each sequence blasted (BLASTN ) at NCBI was compared with the percent similarity scores of the same sequence within the BOLD-IDS (BOLD Identification System) ^20^. Kimura 2-parameter (K2P) congeneric and conspecific variation ^21^, neighbour joining (NJ) and maximum likelihood trees construction were done using the computer program MEGA Version 5.2 ^22^, exported in newick format into FigTree version 1.4.2 ^23^ for visualization and annotation.

## Results 

The read length of COI and 16S rRNA gene sequences obtained were around 681 and 570 bp long, respectively. A total of 48 products were successfully sequenced for both sets of primers ( Table 1). COI gene was sequenced bidirectionally using FishF1 and FishR1 primers while the 16S rRNA gene was sequenced only with the forward primer.

**Table 1.** Species, sampling location and coordinates utilised for the DNA barcoding of the three studied species.

Species	Sampling location	Coordinates		Family	Order	Sample size		Total
		Latitude	Longitude		S	16S	COI	
*C. gariepinus*	Odo-oba	07°28′N	04°08′E	Clariidae	S	7	14	21
*C. gariepinus*	Asejire	07°21′N	04°05′E	Clariidae	P	4	8	12
*S. melanotheron*	Odo-oba	07°28′N	04°08′E	Cichlidae	P	2	4	6
*C. zillii*	Odo-oba	07°28′N	04°08′E	Cichlidae	O	3	6	9
							
						16S	COI	Total
				Total		16	32	48

**Table 2.** Genbank accession numbers for COI and 16S gene sequences for the different fishes.

Sequence no.	COI gene accession no.	16S RNA accession no.	Organism	Specimen voucher no.
Seq1	KX231778	KX243276	*Coptodon zillii*	Coptodon_zilli_odooba_1
Seq2	KX231779	KX243277	*Coptodon zillii*	Coptodon_zilli_odooba_2
Seq3	KX231780	KX243278	*Coptodon zillii*	Coptodon_zilli_odooba_3
Seq4	KX231781	KX243279	*Sarotherodon* *melanotheron*	Sarotherodon_melanotheron_ odooba_4
Seq5	KX231782	KX243280	*Sarotherodon* *melanotheron*	Sarotherodon_melanotheron_ odooba_5
Seq6	KX231783	KX243281	*Clarias gariepinus*	Clarias_gariepinus_odooba_6
Seq7	KX231784	KX243282	*Clarias gariepinus*	Clarias_gariepinus_odooba_7
Seq8	KX231785	KX243283	*Clarias gariepinus*	Clarias_gariepinus_odooba_8
Seq9	KX231786	KX243284	*Clarias gariepinus*	Clarias_gariepinus_odooba_9
Seq10	KX231787	KX243285	*Clarias gariepinus*	Clarias_gariepinus_odooba_10
Seq11	KX231788	KX243286	*Clarias gariepinus*	Clarias_gariepinus_odooba_11
Seq12	KX231789	KX243287	*Clarias gariepinus*	Clarias_gariepinus_asejire_12
Seq13	KX231790	KX243288	*Clarias gariepinus*	Clarias_gariepinus_asejire_13
Seq14	KX231791	KX243289	*Clarias gariepinus*	Clarias_gariepinus_asejire_14
Seq15	KX231792	KX243290	*Clarias gariepinus*	Clarias_gariepinus_asejire_15
Seq16	KX231793	KX243291	*Clarias gariepinus*	Clarias_gariepinus_asejire_16

### COI and 16S sequence analysis

The three fish species sequenced were *C. gariepinus*, *C. zillii* and *S. melanotheron*, the size of each fish sequence obtained (all ≥ 500 bp) was in line with the BOLD-IDS prescription. Only 3 of the 32 samples analysed failed to yield a DNA barcode. All pseudogenes or contaminant sequences were deleted before analysing the sequences. A total of 696 nucleotide sites for the COI gene, and 1049 nucleotide sites for the 16S rRNA gene were observed. Using MEGA 5.2, analysis and exploration of the COI aligned sequences were computed: 347 sites are conserved, 346 are variable (polymorphic) and 225 are parsimony informative. Nucleotide composition analysis revealed the mean frequencies for the complete dataset to be 29.0% for T, 26.6% for C, 26.4% for A and 18.0% for G. The highest percentage G-C at 49.6% was detected in *C. zilli*, while the lowest 42.2% was in *C. gariepinus*. COI sequences contain 347 conserved sites out of 696 (49.86%) bp, 346 variable sites out of 696 (49.71%) bp, 225 parsimony informative sites out of 696 (32.33%) bp and 121 singleton sites out of 696 (17.39%) bp. Nucleotide composition of the 16S rRNA analysis gave a total of 1049 nucleotide sites and revealed the mean frequencies for the dataset to be 269 bp/site (25.64%) conserved, 338 (32.22%) variable, 183 (17.45%) parsimony informative and 155 (14.78%) singletons. The mean frequencies for the complete data were 31.2.0% for T, 22.3% for C, 22.5% for A and 24.0% for G.

Table 3 shows the average number of identical pairs (ii) for COI as 313.33 of which the 1 ^st^, 2 ^nd^ and 3 ^rd^ codons were 556, 206 and 178 respectively. Transitional pairs (si) were found to be lower (si = 25) than transversional pairs (sv = 34). Ratio of si/sv (R) was 0.79 for the dataset. The average number of identical pairs (ii) for 16S rRNA was 153 of which the 1 ^st^, 2 ^nd^ and 3 ^rd^ codons were 154, 152 and 153 respectively. Unlike COI, transversion was the most common substitution detected for all 16S rDNA analysed. In contrast, it was only the transitional pair that was highest in the third codon position whereas transversional pairs were highest at the second codon position (14 and 16 for si and sv, respectively). The average ratio of si/sv (R) was 0.88 for the dataset.

**Table 3.** Directional (16 pairs) nucleotide substitution rate for COI and 16S rRNA genes. All frequencies are average (rounded) over all taxa. ii = identical pairs, Si = transitional pairs, Sv = tranversional pairs, R = Si/Sv and Avg = average.

COI	ii	Si	Sv	R
1 ^st^	556.00	53.00	65.00	0.81
2 ^nd^	206.00	8.00	11.00	0.73
3 ^rd^	178.00	20.00	26.00	0.87
Avg	313.33	27.00	34.00	0.79
16S rRNA	ii	Si	Sv	R
**1 ^st^**	154.00	14.00	16.00	0.88
**2 ^nd^**	152.00	13.00	18.00	0.72
**3 ^rd^**	153.00	16.00	14.00	1.14
**Avg**	153.00	14.33	16.00	0.88

### Species identification by COI and 16S rRNA sequences

Using sequences obtained from the 16 fishes, genetic distances were calculated and compared among the 3 studied species. Table 4 presents the genetic intraspecific variation, which shows that the highest nucleotide divergence was observed in *C. zilli* with nucleotide diversity within the population (π) =0.184 for COI gene, while *S. melanotheron* had the lowest divergence with π=0.065. The highest divergence for 16S rRNA was observed in *C. gariepinus* with π=0.102, while the lowest was *T. zilli* with π=0.019.

**Table 4.** Genetic intraspecific mean variability in *C. gariepinus*, *C. zillii* and *S. melanotheron*. N: the number of sequences; Pi: nucleotide diversity within the population; H: number of different sequences types; K: average number of nucleotide differences within the population.

COI	N	Pi	S.E
*C. gariepinus*	10	0.083	0.007
*C. zillii*	3	0.184	0.015
*S. melanotheron*	2	0.065	0.010
16S rRNA	N	Pi	S.E
*C. gariepinus*	11	0.102	0.004
*C. zillii*	3	0.019	0.006
*S. melanotheron*	2	0.034	0.006

The estimated pairwise genetic distances based on Kimura 2-Parameter Model are presented in Table 3 and Table 6. The lowest nucleotide variation for COI ( Table 5) of 0.17 was observed between *S. melanotheron* and *C. zillii* suggesting a close relationship between these two taxonomic forms. The highest percentage of sequence divergence of 0.49 was found between the *C. gariepinus* and *C. zillii*. The lowest nucleotide variation for 16 S rRNA ( Table 6) of 0.06 (interspecies distance) was observed between *S. melanotheron* and *C. zillii* suggesting a close relationship between these two taxonomic forms. The highest percentage of sequence divergence of 0.61 was found between *C. gariepinus* and *S. melanotheron*.

**Table 5.** COI estimates of pairwise genetic distances between *C. gariepinus*, *C. zillii* and *S. melanotheron* based on Kimura 2-Parameter model. Pairwise conspecific divergence was denoted by the number of base substitutions per site between species (below diagonal) with their corresponding standard error estimate(s) (above the diagonal). Complete deletion of all codon positions (1st, 2nd, 3rd, and Noncoding), were employed in this analysis. All positions. *Genetic distance resulting from intraspecific variation between *C. gariepinus* - S. melanotheron and *C. zillii* – *S. melanotheron*. Mean conspecific divergence, (MCD).

	species	1	2	3	4	5	6	7	8	9	10	11	12	13	14	15	MCD
1.	C.z_0_F_3_COI_F		0.02	0.02	0.03	0.03	0.03	0.04	0.04	0.04	0.03	0.03	0.03	0.03	0.03	0.03	0.181
2.	C.z_0_F_2_COI_F	0.26		0.01	0.02	0.02	0.03	0.02	0.02	0.02	0.02	0.02	0.02	0.02	0.02	0.03	0.181
3.	C.z_0_F_1_COI_F	0.25	0.04		0.02	0.02	0.03	0.02	0.03	0.03	0.02	0.02	0.02	0.02	0.02	0.03	0.181
4.	S.m_O_F_5_COI_F	0.38	0.17*	0.18		0.01	0.02	0.02	0.02	0.02	0.02	0.02	0.02	0.02	0.02	0.02	0.046
5.	S.m_O_F_4_COI_F	0.39	0.18	0.18	0.05		0.02	0.02	0.02	0.02	0.02	0.02	0.02	0.02	0.02	0.02	0.046
6.	C.g_O_F_12_COI_F	0.46	0.30	0.29	0.27	0.26		0.02	0.02	0.02	0.02	0.02	0.02	0.02	0.02	0.02	0.069
7.	C.g_O_F_13_COI_F	0.47	0.28	0.29	0.27	0.25	0.22		0.01	0.01	0.01	0.01	0.01	0.01	0.01	0.01	0.069
8.	C.g_O_F_14_COI_F	0.49	0.28	0.29	0.27	0.27	0.22	0.03		0.00	0.01	0.01	0.01	0.01	0.01	0.01	0.069
9.	C.g_O_F_15_COI_F	*0.49	0.28	0.29	0.28	0.27	0.23	0.02	0.01		0.01	0.01	0.01	0.01	0.01	0.01	0.069
10.	C.g_O_F_6_COI_F	0.46	0.27	0.28	0.26	0.27	0.22	0.03	0.04	0.04		0.00	0.00	0.00	0.00	0.01	0.069
11.	C.g_O_F_7_COI_F	0.45	0.27	0.28	0.26	0.26	0.23	0.03	0.05	0.05	0.01		0.00	0.00	0.00	0.01	0.069
12	C.g_A_F_8_COI_F	0.46	0.27	0.28	0.27	0.27	0.23	0.03	0.05	0.04	0.01	0.01		0.00	0.00	0.01	0.069
13.	C.g_A_F_9_COI_F	0.45	0.27	0.28	0.26	0.26	0.23	0.03	0.05	0.04	0.02	0.01	0.01		0.01	0.01	0.069
14.	C.g_A_F_10_COI_F	0.46	0.28	0.28	0.27	0.27	0.23	0.03	0.05	0.05	0.01	0.02	0.01	0.02		0.01	0.069
15.	C.g_A_F_11_COI_F	0.46	0.28	0.29	0.27	0.27	0.21	0.05	0.06	0.06	0.02	0.03	0.03	0.03	0.02		0.069

**Table 6.** 16S rRNA estimates of pairwise of genetic distances between *C. gariepinus*, *C. zillii* and *S. melanotheron* based on Kimura 2-Parameter model. Pairwise conspecific divergence was denoted by the number of base substitutions per site between species (below diagonal) with their corresponding standard error estimate(s) (above the diagonal). Completed deletion of all codon positions (1st, 2nd, 3rd and Noncoding), gaps and missing data were eliminated and were employed in this analysis. All positions. *Genetic distance resulting from intraspecific variation between *C. gariepinus* - *S. melanotheron* and *C. zillii* – *S. melanotheron*. Mean conspecific divergence, (MCD).

	Species	1	2	3	4	5	6	7	8	9	10	11	12	13	14	15	16	MCD
1.	C.g_A_F_12_16S_F		0.01	0.01	0.01	0.02	0.01	0.01	0.01	0.01	0.01	0.03	0.03	0.03	0.03	0.03	0.03	0.10
2.	C.g_A_F_13_16S_F	0.05		0.00	0.00	0.02	0.00	0.00	0.00	0.00	0.00	0.04	0.02	0.02	0.02	0.02	0.02	0.10
3.	C.g_A_F_14_16S_F	0.05	0.00		0.00	0.02	0.00	0.00	0.00	0.00	0.00	0.04	0.02	0.02	0.02	0.02	0.02	0.10
4.	C.g_A_F_15_16S_F	0.05	0.00	0.00		0.02	0.00	0.00	0.00	0.00	0.00	0.04	0.02	0.02	0.02	0.02	0.02	0.10
5.	C.g_A_F_16_16S_F	0.14	0.10	0.10	0.10		0.02	0.02	0.02	0.02	0.02	0.04	0.03	0.03	0.03	0.03	0.03	0.10
6.	C.g_O_F_6_16S_F	0.06	0.00	0.00	0.01	0.09		0.00	0.00	0.00	0.00	0.04	0.02	0.02	0.02	0.02	0.02	0.10
7.	C.g_O_F_7_16S_F	0.06	0.00	0.00	0.01	0.09	0.00		0.00	0.00	0.00	0.04	0.02	0.02	0.02	0.02	0.02	0.10
8.	C.g_O_F_8_16S_F	0.06	0.00	0.00	0.01	0.09	0.00	0.00		0.00	0.00	0.04	0.02	0.02	0.02	0.02	0.02	0.10
9.	C.g_O_F_9_16S_F	0.06	0.01	0.01	0.01	0.09	0.00	0.00	0.00		0.00	0.04	0.02	0.02	0.02	0.02	0.02	0.10
10.	C.g_O_F_10_16S_F	0.06	0.00	0.00	0.01	0.09	0.00	0.00	0.00	0.00		0.04	0.02	0.02	0.02	0.02	0.02	0.10
11.	C.g_O_F_11_16S_F	0.35	0.42	0.42	0.42	0.50	0.42	0.42	0.42	0.42	0.42		0.05	0.05	0.05	0.05	0.05	0.10
12.	S.m_O_F_4_16S_F	0.27	0.20	0.20	0.20	0.28	0.20	0.20	0.20	0.20	0.20	*0.61		0.00	0.01	0.01	0.01	0.00
13.	S.m_O_F_5_16S_F	0.27	0.20	0.20	0.20	0.28	0.20	0.20	0.20	0.20	0.20	0.61	0.00		0.01	0.01	0.01	0.00
14.	C.z_0_F_1_16S_F	0.27	0.21	0.21	0.21	0.28	0.20	0.20	0.20	0.21	0.20	0.61	0.06	*0.06		0.00	0.00	0.00
15.	C.z_0_F_3_16S_F	0.27	0.21	0.21	0.21	0.28	0.20	0.20	0.20	0.21	0.20	0.61	0.06	0.06	0.00		0.00	0.00
16.	C.z_0_F_2_16S_F	0.27	0.21	0.21	0.22	0.29	0.21	0.21	0.21	0.21	0.21	0.61	0.06	0.06	0.00	0.00		0.00

### Blasting with COI and 16S rRNA genes on NCBI (GenBank) and BOLD-IDS

Comparison of each barcode to the reference sequences submitted previously to BOLD and GenBank resulted in straightforward identification of three species that showed significant species specific similarities based on GenBank and BOLD databases. These databases revealed definitive identity matches in the range of 96%–100% for COI consensus sequences of the three studied species. BLAST results from BOLD database were in agreement with GenBank results in identification of these species, yielding between 99% – 100% identities, except for one sample of *C. zillii*, which had 86% maximum identity in GenBank and no match, which was garnered from BOLD-IDS.

The majority of the GenBank-based identification for all species yielded an alignment E-value of 0.0. GenBank results for *C. gariepinus* ranged between 99% to 100% identity whereas for *S. melanotheron*, the hits were precisely 99% similarity. In the same vein, BOLD-IDS returned hits in the range of 97.84% to 100% species similarity. The database accession numbers and percentage similarity reference sequences with significant species specific similarity obtained from GenBank for all *C. gariepinus* is as follows: APOO432.1 (98%), JQ699203.1 (99%), JQ699203.1 (99%), GU701827.1 (100%), JF894132.1 (99%), HM882821.1 (100%), AP012010.1 (100%), AP012010.1 (100%), AP012010.1 (99%). It also showed significant non-specific similarity (98%) for *C. gariepinus* (query) with *Polypterus seneqalus* (database accession number APOO432.1).

Of the three *C. zillii* individual species barcoded in this study, significant species specific similarity was recorded for two individuals at 99% (GenBank) with accession numbers FJ348137.1 and an insignificant species specific similarity (86%) for one of the species with database accession number JX173760.1. BOLD-IDS also gave significant species specific similarity 99.44% and 99.65% for the *C. zillii* species and no match for one of the samples. These species also showed moderate species specific similarity at 93% and 96% with accession numbers HM882922.1 and HM882911.1 respectively and an insignificant species specific similarity (83%) for one of the species with database accession number HM882922.1.

Thus, when representative COI sequences for the 15 species were compared with existing data, 2 (13.3% of species) shared 100% identity with existing GenBank database entries, 12 (80.0% of species) shared 99% and just one product shared < 97% similarity. Thus, the studied species showed non-ambiguous match categories. GenBank database revealed moderate to definitive identity matches in the range of 93%–99% for consensus sequences of the three studied species with an E-value of zero for all samples. Unlike the COI gene, the GenBank database for *C. gariepinus* samples revealed definitive matches at 99% for all studied species except three sample out of which two samples 1 and 5 ( *C. gariepinus*) were moderately species specific at 93% and 95% similarity, while sample 11 ( *C. gariepinus*) was insignificant at 81% similarity. The accession numbers for all obtained *C. gariepinus* reference sequences are given thus, AP012010.1 (95%), JQ699188.1, JQ699187.1, JQ699184.1 and JQ699185.1 were all (95%) and Q699184.1, JQ699186.1, JQ699184.1, JQ699188.1, JQ699187.1, JQ699185.1 all (99%). For *S. melanotheron*, the percent similarities were species specific significant at 99% for the two species considered in this study with accession number GQ167976.1. It is worth mentioning that it also showed significant non-specific similarity (98%) for *C. gariepinus* (query) with *P. seneqalus* (accession number APOO432.1).

Thus, when representative 16S rRNA sequences for the 16 species were compared with existing data, 13 (81.25% of species) shared 99% identity with existing GenBank database entries, and 3 (18.75% of species) shared < 97% similarity. Two ambiguous or incorrect identifications represented by *P. obscura* were detected and were not included in the final data analysis in MEGA 5.2. Results obtained from similarity search of GenBank confirmed definite species identity for the three studied species but not all the individuals of the two species namely *C. gariepinus* and *C. zillii* produce a significant species specific similarity.

### COI maximum likelihood (ML) and neighbour joining (NJ K2P) trees

**Figure 1.**
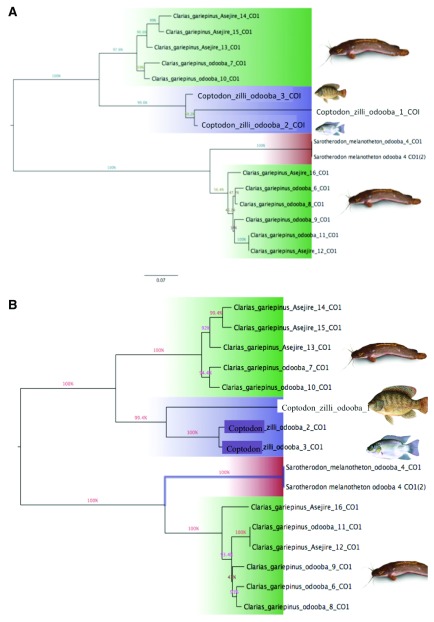
Phylogenetic analysis **A**. Maximum likelihood tree, constructed based on Tamura Neil 3P substitution model. **B**. Neighbour-joining trees based on number of difference model, constructed from COI gene sequences (Bootstrap test was 1000 replicates).

**Figure 2.**
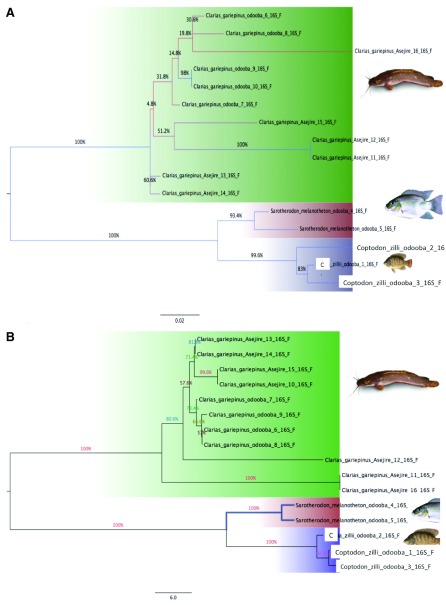
Phylogenetic analysis **A**. Maximum likelihood tree based on Tamura Neil 3P constructed for 16S rRNA gene sequences. **B**. Neighbour-joining trees based on number of difference model constructed from16S rRNA gene sequences. (Bootstrap test was 1000 replicates).

The evolutionary history was inferred using the maximum likelihood (ML) and neighbour joining (NJ) methods based on the Tamura-Nei model and number of difference models, respectively. A full K2P model-based NJ cladogram shows the genetic distance between all specimens that generated a DNA barcode as described above to provide an overview of sequence divergences between all species tested in this study. The consensus tree results computed by the NJ and ML methods are shown in Figure 1 and Figure 2. The distances estimated by the two methods were very similar and the preliminary test with these models built up similar topologies. According to the NJ tree computed for COI sequences ( Figure 1B), the species in the present study were clustered independently within their corresponding genera. This means that closer species in terms of genetic divergence were clustered at the same nodes; However, *C. gariepinus* splits into two clades irrespective of the location. Interestingly, the family Cichlidae did not form an assemblage by clustering together, however, clustered separately within their genera before merging with a 100% bootstrap value. This result is similar to the ML tree obtained to confirm the COI sequence divergence. Moreover, the phylogenetic tree constructed with maximum likelihood method also shows a similar result to the NJ tree ( Figure 1A).

The ML tree based on Tamura Niel 3P was computed for 16S and is presented in Figure 2A. Specimens of the same species did not cluster together as expected. Taxonomic deviation was detected at the species level for all the three studied species and these deviations were reflected at higher levels (genus and family) particularly in the ML tree. Although *C. zillii* is a member of Cichlidae, specimens were clustered separately with species of *C. gariepinus* and *S. melanotheron*, under separate nodes; however, the low bootstrap values in the upper portion of the tree suggest that the topology of the consensus tree is unreliable. The reverse was the case as indicated in Figure 2B, a close inspection of the K2P NJ tree revealed that distinction existed between two cichlids *S. melanotheron* and *C. zillii* relative to the ML tree. These species clustered separately within their own genera and were unambiguously separated. In other words, all specimens of the same species were clustered together. This also applied to *C. gariepinus*, although in each of these cases, 10 samples of *C. gariepinus* were clearly separated at the species level but under two different clades. They clustered under the same family and formed two separate clusters on the NJ tree.

## Discussion

The DNA barcode approach provides additional important data for the precise identification and classification of diverse biodiversity ^24^. The COI and 16S rRNA markers used in our study were useful for the identification of the studied fish species. Our observations in this study show that phylogenetically, COI sequences effectively clustered most conspecific and congeneric species. This was also observed in similar studies in Australian fishes ^25^, Canadian freshwater fishes ^26^, freshwater fishes from southeastern Nigeria ^13, 14^, Indian catfishes ^27^ and freshwater fishes from Indonesia ^28^. Earlier studies have described the utility of COI and 16S rRNA as candidate DNA markers ^29^. Our results show that mtDNA COI has a higher degree of DNA variability than mt 16S rRNA and importantly, is widely used for fish identification ^30^. Furthermore, recent studies have revealed that the 16S rRNA failed to distinguish closely related species due to their lower genetic variability ^31^. We find that using the ML tree and the genetic pairwise distance matrix, the 16S rRNA failed to distinguish conspecific species in the sampled *Tilapia* populations, as shown by low bootstrap values in the phylogenetic tree. The low power of 16S rRNA can be attributed to the paucity of informative sites compared with mtDNA COI. Cawthorn and co-workers using the 16S rRNA were also not able to distinguish 53 commercial fish species ^31^. However, species identification based on mtDNA COI was unambiguous, our results suggest that COI sequence provides sufficient genetic variability for all studied species especially sampled populations of *C. zillii* and *S. melanotheron*.

The mean genetic K2P genetic distances of COI were similar between intraspecific and interspecific species but different at the confamilial taxonomic level suggesting the absence of a barcoding gap, an observation also made by Zou *et al.*, ^32^. A study on publicly available sequences of marine and freshwater fishes available from the Barcoding of Life Database also reported a paucity of barcoding gap in COI ^1, 20^. The cichlid populations showed varying degrees of introgression and hybridization with respect to the clariid family examined in this study. An explanation for this may be that the infrequent mating of closely related species may bring about hybridization of offspring’s, which for maternally inherited mitochondrial genes, may result in phylogenetic paraphyly ^33– 35^. There have been several reports of autochthonous hybridization between closely related species occasioned by human-induced-changes to local habitats. This may suggest parapatric speciation between *C. zilli* and *T. guineensis* in many rivers where they co-exist ^36^. Most of the comparisons done in this study were within the 3% score mark and are in line with the suggestion of Wong and Hanner ^37^, except for one species of sampled *C. zillii,* which returned no match. Thus, it was described further as unambiguous as BOLD-IDs suggested that it could either be *C. zillii* or *T. guineensis*. Within a conspecific distance of less than 2%, BOLD-IDS validates its identification search of a species query sequence. This is usually only when the species in the BOLD-IDS database contains at the least, three barcoded specimens ^22^. Low (86%) match was also recorded with *C. zillii* sequences in GenBank. Thus, it is strongly suspected that this result is insignificant and that *C. zillii* sequences stored in both the BOLD and GenBank databases were originally specimens of *T. guineensis* or hybridized. Therefore, it can be interpreted that *T. guineensis* may be actually *C. zillii* with regards to NCBI BLASTN search.

The hallmark of barcode analysis is to delineate species boundaries. This is in conformance with our observed results utilising the NJ trees, as there was an obvious phylogenetic signal in COI sequence data ^38^. All NJ trees for both markers resolved species-specific clades that were supported by moderate to high bootstrap values. *C. gariepinus* did not form a distinct clade even when they clustered together in both COI (NJ and ML trees) and 16S rDNA (only NJ tree). The clustering of *C. gariepinus* ( Figure 1A) and *C. gariepinus* ( Figure 1B) in different lineages is due to phylogenic separation. This has been confirmed in a similar study carried out by Funk and Omland ^35^ and is congruent with the data derived from this study. In their study, geographical separation during early stages of their evolution resulted in *C. macrocephalus* and *C. batrachus* in one lineage and *C. gariepinus* in another lineage; *C. macrocephalus* and *C. batrachus* are native Asian catfish while *C. gariepinus* is of African origin. Geographic differentiation is therefore apparent for the *C. gariepinus* species, with one clade comprising the Oodoba (O) individuals and another comprising the Asejire (A) individuals. Our observations from results from the K2P/NJ tree reveal a clustering together of similar species. These results are in line with present taxonomic classifications of the three fishes studied.

Another anomalous observation in the ML tree of the 16S rRNA is that of *S. melanotheron* clustered with species *C. zillii*. This indicates deviations at the genus level and may indicate a shared haplotype although the bootstrap values obtained were significant. This aberration was not noticed with the COI as it clustered *S. melanotheron* specimens together. Therefore, the species did not cluster independently within their corresponding genera. The monophyly of exhibited by COI for the two cichlids was supported by 16S rRNA, but the position of the clade formed by *C. zillii* in relationship with *S. melanotheron* is dependent on the molecular marker selected for the phylogenetic analysis. The data reveal a close relationship between *C. zillii* and the clade formed by *S. melanotheron*, which is not surprising taking into consideration that they are both from the same family. The NJ method, which differentiated the 16S rRNA sequences without ambiguity compared with the ML tree, which failed to produce similar result and clustered the species erroneously indicating that they possess a shared haplotype. Thus, the 16S tree generated by the ML tree method was unable to separate the nucleotide sequences of three studied species. Consequently, this ambiguity can be resolved using more markers such as microsatellites, which have proved useful in species delimitation ^39^.

The report emanating from this study was not wholly congruent with the phenomenon of increasing evolutionary divergences in taxonomic levels within and among species. This is supported by a case study carried out by Zou *et al.*, ^33^ on Neogastropoda where the barcoding gap between levels of intraspecific variation and interspecific divergence does not exist in either analysis of COI or 16S rDNA sequences. This could be attributed probably to very limited sample sizes employed in this study at each taxonomic level ^28^. However, despite this obvious limitation, one interesting observation is the genetic relatedness between *S. melanotheron* and *C. zillii*. The Tilapiine fishes, *S. melanotheron* and *C. zillii*, could not be separated by the 16S marker. This was because it lacked resolution in species differentiation, a key weakness of the marker. This was despite similar mean K2P-distances obtained at multiple taxonomic levels suggesting the lack of a barcoding gap. However, COI was able to clearly separate the fish species ruling out introgression of the species as responsible for this limitation. Our observations demonstrate the need for precise species identification in the generation of any barcode library. Thus, classification of both Clariid and Cichlid species from South West Nigeria has benefited from phylogenetic analysis using mitochondrial ribosomal genes as markers.

## Conclusion

Although Nigeria is not a major fishing nation, focus on freshwater and marine conservation, and studies about the early life history of all fishes are essential for the management of its aquatic resources. Therefore, identification of fishes based on morphological characteristics should be complemented with molecular methods of identification. In addition, other markers such as nuclear DNA can be used in identification and estimating fish population. Our results clearly underlie the efficiency of DNA barcoding in the identification of the three species in Southwest Nigeria. Proper species identification is important in fish conservation and management. Thus, DNA barcoding will be a useful tool for monitoring of conservation in fisheries management programmes in Nigeria.

## Data availability

The data referenced by this article are under copyright with the following copyright statement: Copyright: © 2016 Falade MO et al.

Raw sequence data for the samples reported here can be found in GenBank under accession numbers: KX231778, KX231779, KX231780, KX231781, KX231782, KX231783, KX231784, KX231785, KX231786, KX231787, KX231788, KX231789, KX231790, KX231791, KX231792, KX231793, KX243276, KX243277, KX243278, KX243279, KX243280, KX243281, KX243282, KX243283, KX243284, KX243285, KX243286, KX243287, KX243288, KX243289, KX243290, KX243291.
