# Supplementary material for: DNA barcoding of Clarias gariepinus, Coptodon zillii and Sarotherodon melanotheron from Southwestern Nigeria
Source: F1000Res. 2016 Jun 8;5:1268. [Version 1] doi: 10.12688/f1000research.7895.1 (PMC5133684; doi:10.12688/f1000research.7895.1)
Supplement: Peer review history

# Peer review: Referee response for version 1

**DOI:** 10.5256/f1000research.8499.r16270

**Published:** 2016-11-29

**Authors:** Shadi Shokralla

**Affiliations:** 1 Department of Integrative Biology, Biodiversity Institute of Ontario, University of Guelph, Guelph, ON, Canada

The current MS is a good case study showing the efficiency of the DNA barcoding in study the diversity of different groups of organisms.

 I have some comments to improve the MS:

- The sample size is very small to conduct any diversity measure.
- All gene names should be italic.
- How many mg was the starting material for DNA extraction?
- I was expecting to see more taxonomic identification in this work. Also it will be great if the authors can add some information about the habitation of these tested species.
- I suggest to the author to do some phylogenetic comparisons between CO1 and 16S not only with the species tested in this work but also other species of the same genus or other species of the same family to enrich the study more.

I have read this submission. I believe that I have an appropriate level of expertise to confirm that it is of an acceptable scientific standard.

# Peer review: Referee response for version 1

**DOI:** 10.5256/f1000research.8499.r14760

**Published:** 2016-07-06

**Authors:** Dora Davies

**Affiliations:** 1 Faculty of Natural Sciences, National University of Salta, Salta, Argentina

The authors contribute to the knowledge of species of freshwater fish of interest in Nigeria, whose diversity is poorly known, to using molecular tools that complement the difficult morphological studies by the shortage of taxonomists and lack of reliable morphological characters for discriminating species.

 Confirm the usefulness of COI, especially, to discriminate phylogenetically close taxa, although there is some overlap between the intraspecific and interspecific variation, which partially obscures the delimitation of species. The number of samples involves a remarkable effort, aimed at improving the accuracy of the results.

 Minor suggestions are given:

 In the expression " *Tilapia (Coptodon)*" *Coptodon* seems a subgenre of *Tilapia*. One could say “ *Coptodon*, previously included in Tilapia”

 In the third paragraph of the introduction, second line, should write the name of the genus *Clarias* and not its abbreviation, C., because it could be confused with the name of the other gender that also begins with C, *Coptodon*.

 In the second paragraph, second line of PCR and DNA sequencing item, add a space between 10 and

 μL after point.

 In the body of Table 1 labels the fifth row are not necessary. You should delete the row.

 Table 4 does not include data for H: number of different types sequences; K: average number of nucleotide differences within the population. Add the data in the table.

 In the paragraphs where the authors compare the sequences of COI and 16S rRNA with those published in GenBank, the term species use instead of specimens. Then replace species by specimens in all cases.

 Figure 1 and 2: numbers are unclear and are above the lines.

I have read this submission. I believe that I have an appropriate level of expertise to confirm that it is of an acceptable scientific standard.
